# Gastric cancer-secreted galectin-1 promotes peritoneal mesothelial-mesenchymal transition to prime peritoneal metastasis soil

**DOI:** 10.1016/j.isci.2026.115908

**Published:** 2026-04-27

**Authors:** Chuanjiang Huang, Zhipeng Jiang, Xiaoqin Shen, Qingzhu Ding, Huina Wang, Zhiyi Cheng, Guiyuan Liu, Xiaojun Zhao, Xiaolan You

**Affiliations:** 1 Department of Gastrointestinal Surgery, The Affiliated Taizhou People’s Hospital of Nanjing Medical University, Taizhou 225300, Jiangsu, China; 2 Department of Anesthesiology, The Affiliated Taizhou People’s Hospital of Nanjing Medical University, Taizhou 225300, Jiangsu, China

**Keywords:** classification description, health sciences, medicine, oncology, natural sciences, biological sciences, cancer systems biology, cancer

## Abstract

Peritoneal metastasis (PM) is the most common cause of death in gastric cancer (GC) patients. Targeting the molecular mechanisms of PM in GC could reduce its incidence and improve clinical outcomes. Using clinical specimen analysis, lentivirus transfection, western blotting, RT-qPCR, immunofluorescence, cell adhesion assays, and a GC peritoneal metastasis (GCPM) animal model, we explored galectin-1’s role in promoting peritoneal MMT (mesothelial-mesenchymal transition) and enhancing PM through *in vivo* and *in vitro* studies. Galectin-1 in the GC microenvironment reprograms peritoneal mesothelial cells to facilitate PM, with its high expression in GC tissues being a key factor for PM. Galectin-1-induced peritoneal MMT through the TGF-β/Smad signaling pathway is an important mechanism for GCPM, offering a potential target for GC treatment.

## Highlights

• Gal-1 upregulation strongly correlates with peritoneal MMT and GCPM
• GC-secreted Gal-1 drives peritoneal MMT via the TGF-β/Smad signaling pathway
• Gal-1 promotes GC cell adhesion to peritoneal cells
• TGF-β/Smad blockade reverses Gal-1-induced peritoneal MMT and inhibits GCPM

## Graphical abstract

**Figure:**
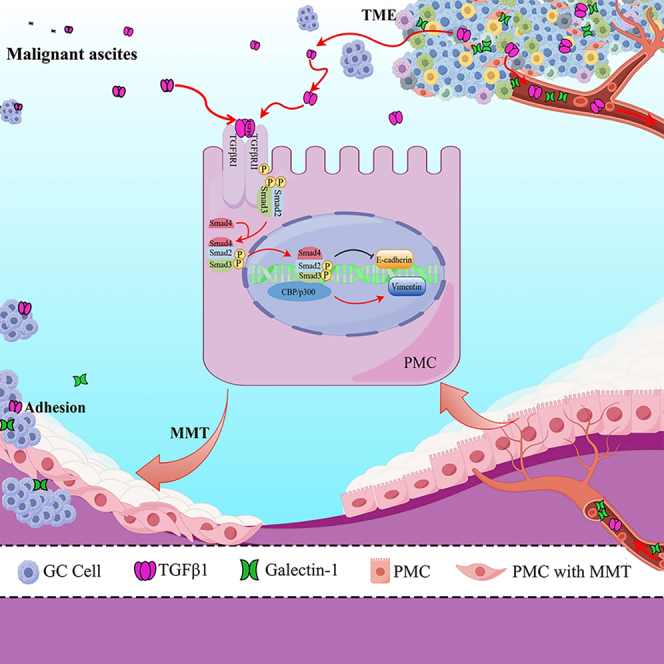

## Introduction

Gastric cancer (GC) is a common malignancy within the gastrointestinal tract and is considered an aggressive disease with a dismal prognosis.^1^ The early stage of GC typically presents no symptoms, and approximately 80%–90% of GC patients are diagnosed at an advanced stage, accompanied by a poor clinical prognosis.^2^ Peritoneal metastasis (PM) is the most prevalent form of metastasis in GC patients, accounting for 53%–60% of all manifestations of metastasis, and is a vital cause of death among these patients.^3^ Once gastric cancer peritoneal metastasis (GCPM) occurs, the median survival time is only 3–6 months, and the 5-year survival rate is only 2%.^4^ Even after radical surgery for GC, up to 50% of advanced GC patients develop PM, leading to an extremely poor prognosis.^4^^,^^5^ The “seed and soil” theory has gained widespread acceptance as a classical theory of PM.^6^^,^^7^ Nevertheless, a greater emphasis in research has been placed on the “seeds,” with insufficient attention given to the impact of alterations in the “soil” (i.e., the peritoneum) on GCPM. Elucidating the underlying molecular mechanisms is of great significance for improving clinical diagnosis and treatment strategies, as well as enhancing the survival outcomes of GC patients.

Over the past few centuries, tumor biologists have focused on investigating the molecular biological characteristics of tumor cells, neglecting the decisive role of the tumor microenvironment in tumorigenesis and development. The tumor microenvironment comprises tumor cells, stromal cells, chemokines, cytokines, etc., facilitating tumor growth and metastasis.^8^ Stromal cells include cancer-associated fibroblasts (CAFs), mesenchymal stem cells, immune cells, neuroendocrine cells, and endothelial cells, while cytokines and chemokines are important soluble factors in the microenvironment.^9^ The combination of stromal cells, cytokines, and chemokines within the tumor microenvironment promotes tumor angiogenesis,^10^ enhances the directional migration, invasion, and metastasis capabilities of tumor cells, manipulates immune cells through immune editing, subverts immune surveillance, and ultimately leads to immune escape, thereby promoting tumor growth and distant metastasis. Hence, targeted therapy for the tumor microenvironment has attracted extensive attention among scholars.^11^^,^^12^^,^^13^

CAFs within the GC microenvironment exhibit high expression of galectin-1 (Gal-1, encoded by *LGALS1*),^14^ and galectin-1 can promote homotypic cell aggregation, enhance the adhesion of tumor cells to the extracellular matrix, facilitate tumor cell migration and invasion, and induce tumor angiogenesis.^15^ These four effects cover the critical steps of PM in GC: cancer cells escape from the primary tumor, survive freely in the abdominal cavity, attach to the distant peritoneum, and invade subperitoneal tissues to proliferate through neovascularization. Therefore, we infer that galectin-1 is an important factor regulating GCPM.

Tumor metastasis is a multifactorial and multistep cascade event involving the behavior of tumor cells and the specificity of target tissues. In the peritoneal environment, the mesothelial layer composed of peritoneal mesothelial cells (PMCs) constitutes the initial barrier against bacterial and tumor invasion. During tumor metastasis, PMCs can undergo mesothelial-mesenchymal transition (MMT) to facilitate tumor peritoneal implantation; however, the specific mechanisms involved remain unclear. Kaplan reported that prior to tumor cell metastasis, distant target tissues or organs, termed premetastatic niches (PMNs), often undergo adaptive alterations that favor tumor cell growth.^16^ The formation of PMNs is believed to be induced by tumor-secreted cytokines and exosomes. Our previous studies demonstrated that galectin-1 can regulate the TGF-β/Smad signaling pathway, facilitate epithelial-mesenchymal transition (EMT) in GC cells, and enhance their invasion and migration capabilities.^14^ However, the effect of galectin-1 on PMCs remains poorly understood. Furthermore, while previous studies have predominantly focused on the impact of CAF-derived galectin-1 on GC cells,^17^^,^^18^ there is a notable lack of knowledge regarding the “dual roles” of galectin-1; specifically, the intracellular functions after being internalizated by target GC cells, and the alterations in the biological behavior of GC cells that overexpress galectin-1. The aim of this study was to uncover the regulatory effect of galectin-1 on PMCs and its role in the GCPM using clinical specimens and *in vivo/in vitro* experiments.

## Results

### Galectin-1 expression in GC is related to peritoneal MMT and metastasis

**Figure 1:**
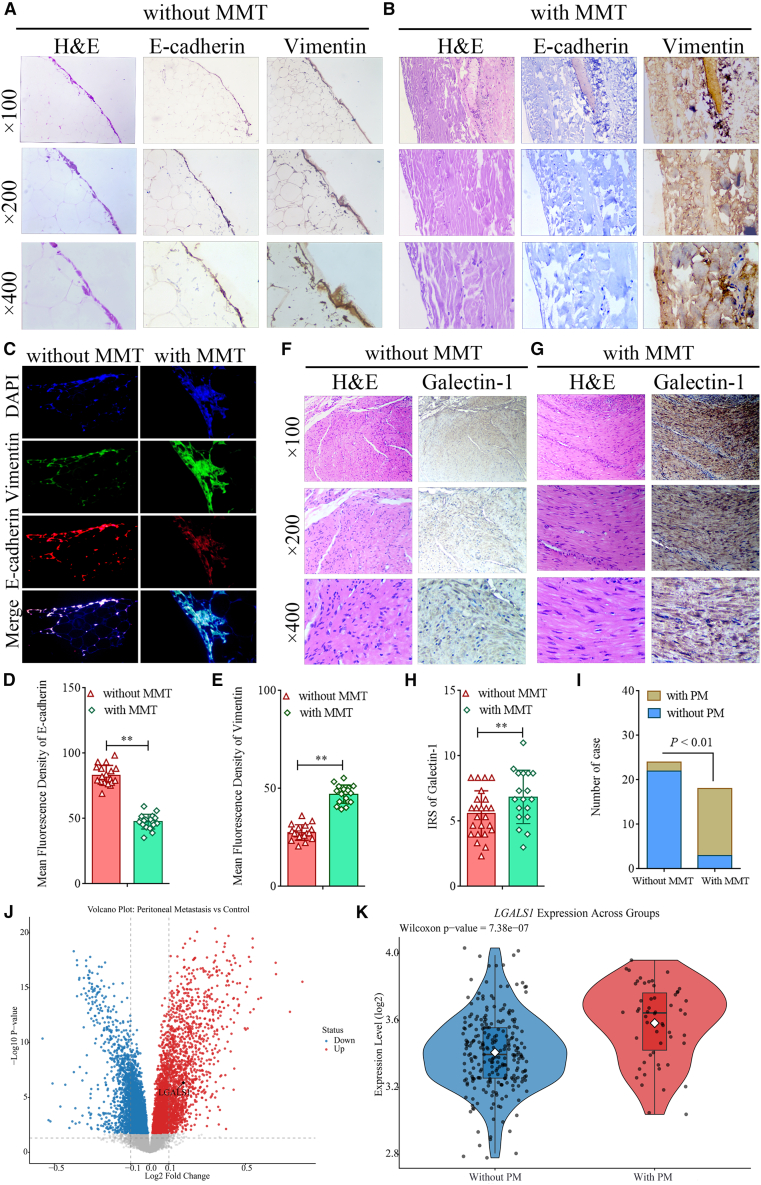
Galectin-1 expression in GC is associated with peritoneal MMT and PM (A and B) Representative H&E and IHC staining for E-cadherin and vimentin in peritoneal tissues without or with MMT, at ×100, ×200, and ×400 magnification. (C) Immunofluorescence co-staining of E-cadherin, and vimentin to assess MMT status. (D and E) Quantification of mean fluorescence intensity for E-cadherin and vimentin (*n* = 6). (F and G) Representative H&E and IHC staining for galectin-1 in GC tissues without or with MMT, at ×100, ×200, and ×400 magnification. (H) Immunoreactive score (IRS) of galectin-1 expression (*n* = 42). (I) Comparison of the PM state in patients without (*n* = 24) or with (*n* = 18) peritoneal MMT. (J) Volcano plot of differential gene expression from GSE62254 (*n* = 300), analyzed by limma; *LGALS1* is highlighted as upregulated in PM. (K) Violin plot of *LGALS1* expression in the group without PM vs. with PM group. Data are represented as mean ± SD.∗*p* < 0.05, ∗∗*p* < 0.01.

To clarify the relationship between PM and the peritoneal MMT in patients with GC, E-cadherin (encoded by *CDH-1*) and vimentin (encoded by *VIM*) proteins were analyzed in the peritoneal tissues of 42 GC patients through immunohistochemistry (IHC). Our study revealed a decreased expression of E-cadherin and elevated expression of vimentin in the peritoneal tissues of 18 GC patients, indicating the occurrence of peritoneal MMT. Representative images of peritoneal tissue without MMT are shown in Figure 1A, and Figure 1B presents images of peritoneal tissue with MMT. Immunofluorescence (IF) was utilized to quantify the E-cadherin and vimentin proteins in peritoneal tissues. Figure 1C shows representative images of the IFs, and Image-Pro Plus software was used to analyze the mean fluorescence density of each protein. The statistical results indicated that the group with MMT had significantly lower E-cadherin (Student’s *t* test, *p* < 0.01, Figure 1D) and significantly higher vimentin (Student’s *t* test, *p* < 0.01, Figure 1E) than the group without MMT. Subsequently, hematoxylin and eosin (H&E) and IHC were employed to detect the expression of galectin-1 in the GC tissues of patients without or with peritoneal MMT (Figures 1F and 1G). The IRS results revealed that galectin-1 in the GC tissues of the group with peritoneal MMT was significantly higher than that in the group without MMT (Student’s *t* test, *p* < 0.01, Figure 1H). These 42 patients were followed up for more than 5 years until December 2023. The clinical follow-up results revealed that 2 of 24 patients without MMT had PM, whereas 15 of 18 patients with MMT had PM, and the difference between the two groups was highly statistically significant (chi-square test, *p* < 0.01, Figure 1I). To further validate the regulatory role of *LGALS1* in GCPM, we retrieved the GC gene expression dataset GEO: GSE62254 (*n* = 300) from the Gene Expression Omnibus database. Based on clinical follow-up data, samples were stratified into with PM group (*n* = 54) and without PM group (*n* = 246). Differentially expressed genes were identified using the linear models implemented in the limma package. Probe-level data were aggregated into a gene-level expression matrix according to the GPL570 platform annotation file. Compared with the group without PM, *LGALS1* was among the significantly upregulated genes in the group with PM (Figure 1J). Notably, *LGALS1* expression was markedly elevated in the group with PM (Wilcoxon rank-sum test, *p* = 7.38 × 10^−7^, Figure 1K), suggesting that *LGALS1* may serve as a potential regulator in GCPM.

### Galectin-1 promotes MMT in peritoneal mesothelial cells

**Figure 2:**
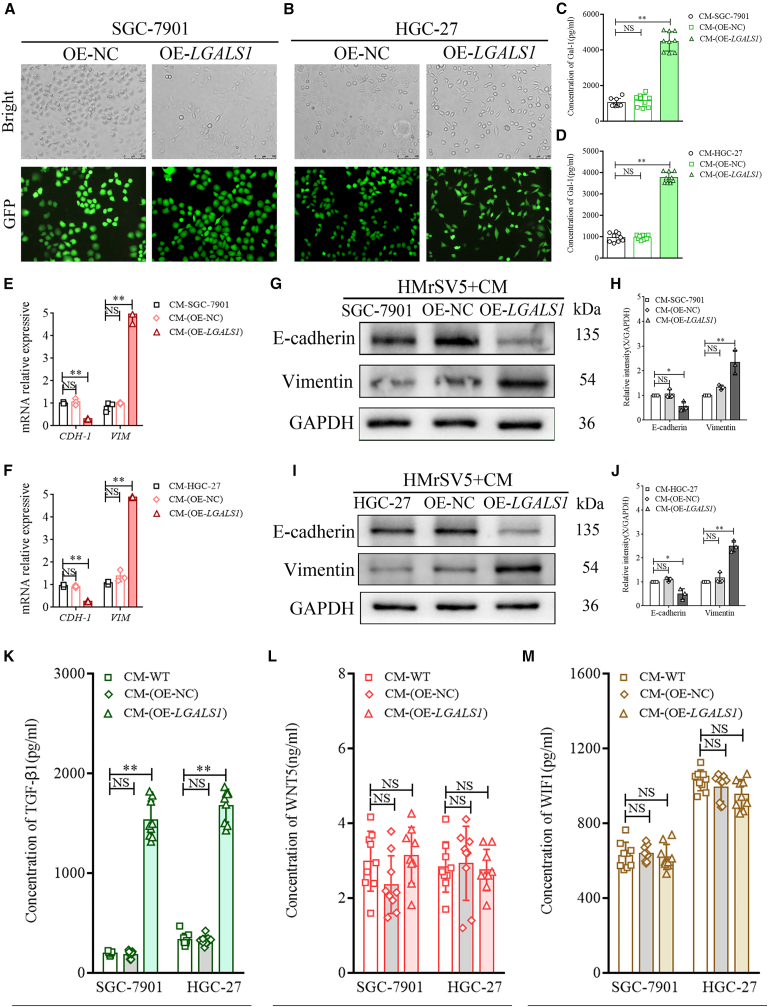
Galectin-1 promotes MMT in HPMCs and upregulates TGF-β1 expression (A and B) Representative bright-field and GFP images of *LGALS1* overexpression (OE-*LGALS1*) and negative control (OE-NC) SGC-7901 (A) and HGC-27 (B) cells (Scale bars, 100 μm). (C and D) ELISA analysis of galectin-1 in CM from WT, OE-NC, and OE-*LGALS1* SGC-7901 and HGC-27 cell lines (*n* = 3). (E and F) RT-qPCR analysis of *CDH1* and *VIM* mRNA in HMrSV5 cells treated with CM from SGC-7901 and HGC-27 cell lines (*n* = 3). (G–J) WB confirmed E-cadherin and vimentin expression in HMrSV5 cells treated with CM from SGC-7901 and HGC-27 cell lines (*n* = 3). (K–M) ELISA analysis of TGF-β1 (K), WNT5 (L), and WIF1 (M) in CM from SGC-7901 and HGC-27 cell lines (*n* = 3). Data are represented as mean ± SD.∗*p* < 0.05. ∗∗*p* < 0.01, NS, *p* > 0.05.

To reveal the mechanism by which galectin-1 promotes peritoneal MMT and enhances PM of GC cells, we constructed a *LGALS1* overexpression lentivirus and generated stably transfected SGC-7901 and HGC-27 cell lines, namely OE-*LGALS1* and OE-NC. Representative bright-field and fluorescence images of stably transfected SGC-7901 and HGC-27 cells are shown in Figures 2A and 2B. ELISA was used to detect galectin-1 expression in the conditioned medium (CM) of SGC-7901 and HGC-27 cells with different *LGALS1* expression levels. The results showed no significant difference in galectin-1 levels between the wild-type control group and the OE-NC group (NS, *p* > 0.05), whereas galectin-1 concentration in the OE-*LGALS1* group was significantly higher than that in the two control groups (one-way ANOVA, *p* < 0.01; Figures 2C and 2D).

To further elucidate the promotion of MMT by galectin-1 in human peritoneal mesothelial cells (HPMCs), we collected CM from the above GC cell lines to treat human peritoneal mesothelial cell line HMrSV5 and detected the expression of *CDH-1* and *VIM* mRNAs in the treated HMrSV5 cells via RT-qPCR. Compared with the CM-SGC-7901 and CM-OE-NC groups, CM-OE-*LGALS1* significantly inhibited *CDH1* expression and increased *VIM* expression (one-way ANOVA, *p* < 0.01, Figure 2E), while no significant difference was observed between the two control groups. The results of HMrSV5 cells treated with CM from the HGC-27 cell line were consistent with those of CM from SGC-7901 cells (Figure 2F).

Then, we used western blotting (WB) to detect the expression of E-cadherin and vimentin in HMrSV5 cells treated with different CMs. The results demonstrated that E-cadherin protein levels were significantly lower and vimentin levels significantly higher in HMrSV5 cells treated with CM-OE*-LGALS1* from SGC-7901 than in cells treated with CM-SGC-7901 and CM-OE-NC (one-way ANOVA, *p* < 0.05 and *p* < 0.01, Figures 2G and 2H). Similar results were observed in HMrSV5 cells treated with CM derived from HGC-27 cell lines, consistent with the findings in SGC-7901 cells (Figures 2I and 2J).

### Galectin-1 promotes MMT in HPMCs through the TGF-β/Smad signaling pathway

To further explore the molecular mechanism through which galectin-1 promotes MMT in HPMCs, we utilized enzyme-linked immunosorbent assay (ELISA) to detect the expression of TGF-β1, WNT5, and WIF1 in the CM of GC cell lines. Our results revealed that the TGF-β1 level in CM from SGC-7901 OE-*LGALS1* cells was significantly higher than that in CM-SGC-7901 and CM-OE-NC cells; the TGF-β1 expression in the CM of HGC-27 cells was consistent with that in SGC-7901 CM (one-way ANOVA*,* all *p* < 0.01, Figure 2K). However, the expression of WNT5 and WIF1 showed no significant difference between the CM-OE-*LGALS1* groups and other groups (one-way ANOVA*,* all *p* > 0.05, Figures 2L and 2M).

**Figure 3:**
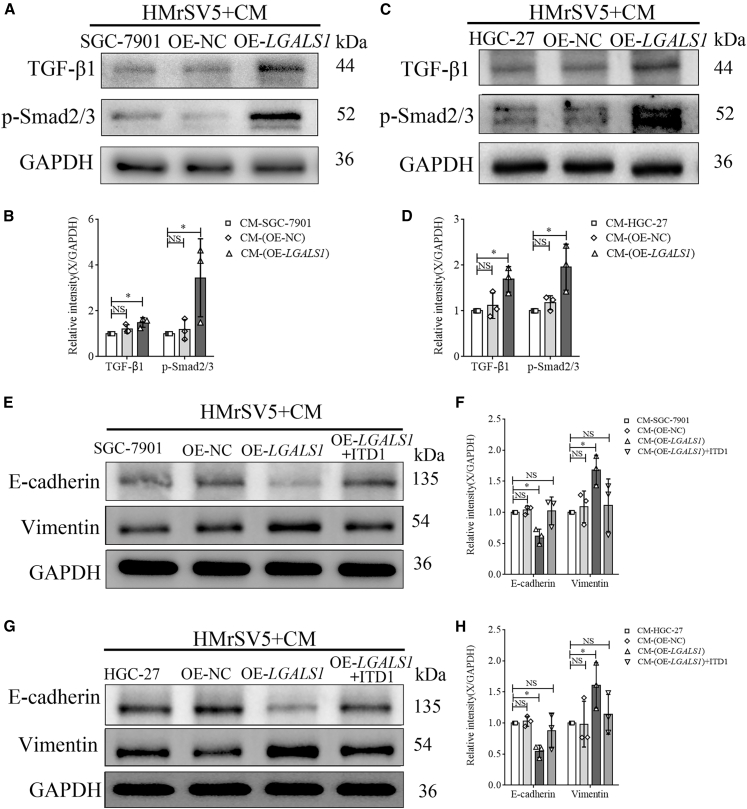
Galectin-1 promotes MMT in HPMCs through the TGF-β/Smad signaling pathway (A–D) WB analysis of TGF-β1 and *p*-Smad2/3 in HMrSV5 cells treated with CM from SGC-7901 cells (A and B) and HGC-27 cells (C and D) with different *LGALS1* expression levels (*n* = 3). (E–H) WB confirmed E-cadherin and vimentin expression in HMrSV5 cells treated with CM from SGC-7901 cells (E and F) and HGC-27 cells (G and H) with different *LGALS1* expression levels or CM-OE-*LGALS1* supplemented with ITD1. Data are represented as mean ± SD. ∗∗*p* < 0.01, NS, *p* > 0.05.

WB was subsequently used to detect the expression of TGF-β1 and *p*-Smad2/3 in HMrSV5 cells treated with CM from SGC-7901 cells with different *LGALS1* levels (Figure 3A). Our study revealed that TGF-β1 and *p*-Smad2/3 expression in HMrSV5 cells treated with CM-OE-*LGALS1* was significantly higher than that in CM-SGC-7901 and CM-OE-NC cells (one-way ANOVA, all *p* < 0.05, Figure 3B). We repeated this study with HGC-27 cells, and the results were consistent with those of SGC-7901 cells (Figures 3C and 3D).

To further clarify that galectin-1 can activate the TGF-β/Smad signaling pathway, CM from SGC-7901 (OE-*LGALS1*, OE-NC, and wild-type) and ITD1 (a specific inhibitor of the TGF-β/Smad pathway) was used to treat HMrSV5 cells. Next, we detected the expression of E-cadherin and vimentin in HMrSV5 cells from each group via WB (Figure 3E). We found that both the decrease in E-cadherin and increase in vimentin in HMrSV5 cells treated with CM-OE-*LGALS1* (one-way ANOVA, all *p* < 0.05) could be reversed by ITD1 (Student’s *t* test, all *p* > 0.05, Figure 3F). We repeated this study with CM from HGC-27 cells, and the results were consistent with those from SGC-7901 cells (Figures 3G and 3H). These findings suggest that galectin-1 promotes MMT in PMCs through the TGF-β/Smad signaling pathway.

### Activation of the TGF-β/Smad signaling pathway promotes peritoneal MMT

**Figure 4:**
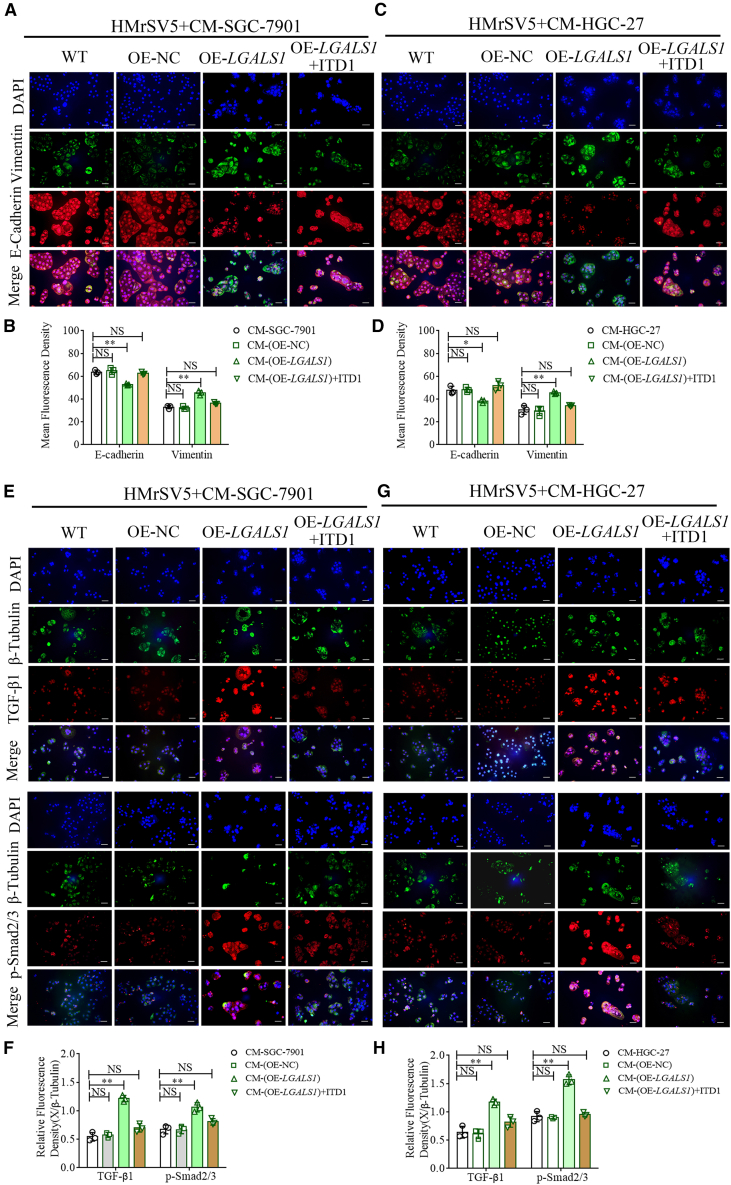
Activation of the TGF-β/Smad signaling pathway promotes MMT in HPMCs (A–D) Representative immunofluorescence images of E-cadherin and vimentin in HMrSV5 cells treated with CM from SGC-7901 cells (A and B) and HGC-27 cells (C and D) with different *LGALS1* expression levels or CM-OE-*LGALS1* supplemented with ITD1 (Scale bars, 50 μm) (*n* = 3). (E–H) Representative immunofluorescence images of TGF-β1 and *p*-Smad2/3 in HMrSV5 cells treated with CM from SGC-7901 cells (E and F) and HGC-27 cells (G and H) with different *LGALS1* expression or CM-OE-*LGALS1* supplemented with ITD1 (Scale bars, 50 μm) (*n* = 3). Data are represented as mean ± SD.∗*p* < 0.05, ∗∗*p* < 0.01, NS, *p* > 0.05.

Immunocytochemistry was employed to verify the relationship between the activation of the TGF-β/Smad signaling pathway and peritoneal MMT. The fluorescence intensity of E-cadherin in HMrSV5 cells treated with CM-OE-*LGALS1* from SGC-7901 cells was decreased, and that of vimentin was increased (one-way ANOVA, all *p* < 0.01, Figures 4A and 4B), whereas CM-OE-*LGALS1* supplemented with ITD1 restored the expression of E-cadherin and vimentin (Student’s *t* test, all *p* > 0.05, Figure 4B). We also replicated the experiment with CM from HGC-27 cells, and the results were in accordance with those from SGC-7901 cells (Figures 4C and 4D). We subsequently detected the expression of TGF-β1 and *p*-Smad2/3 in HMrSV5 cells treated with CM (Figure 4E); the relative fluorescence intensities of TGF-β1 and *p*-Smad2/3 in HMrSV5 cells treated with CM-OE-*LGALS1* from SGC-7901 cells were significantly increased(one-way ANOVA, all *p* < 0.01, Figure 4F), and the differential expression of proteins induced by *LGALS1* was restored by ITD1 (Student’s *t* test, all *p* > 0.05, Figure 4F). The results of the replication experiment with CM from HGC-27 cells were consistent with those with CM from SGC-7901 cells (Figures 4G and 4H).

**Figure 5:**
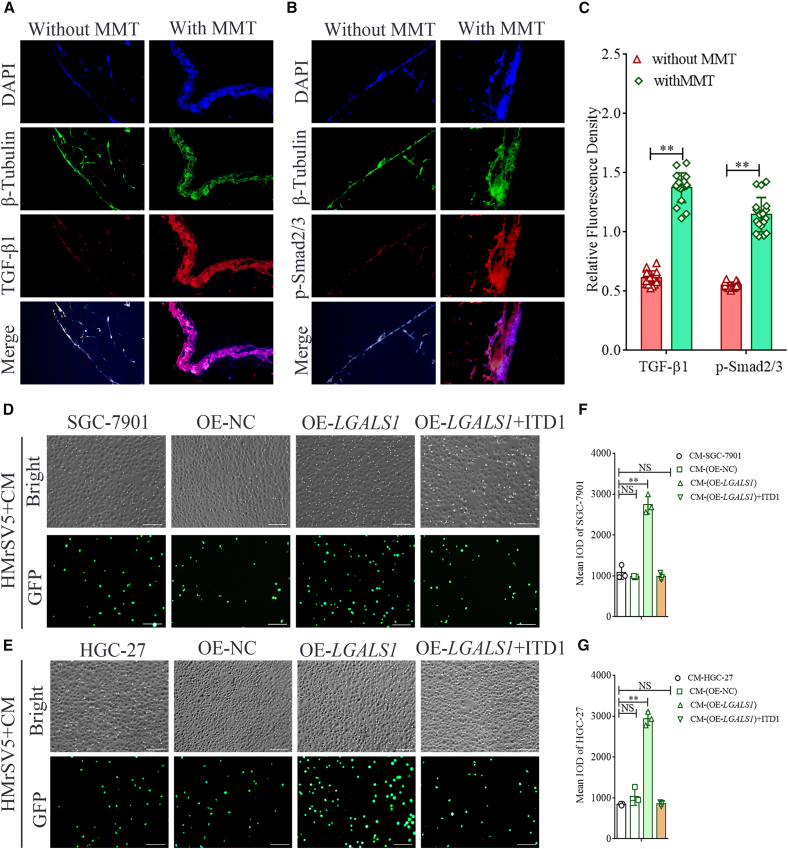
TGF-β/Smad signaling pathway is activated in peritoneal tissues undergoing MMT, and galectin-1 enhances GC cell adhesion to HPMCs via this pathway (A–C) Representative images of immunofluorescence for TGF-β1 (A) and *p*-Smad2/3 (B) in peritoneal tissues without or with MMT (×400 magnification). (C) Comparison of the relative fluorescence density of TGF-β1 and *p*-Smad2/3 in peritoneal tissues without or with MMT (*n* = 6). (D and E) GC cells incubated with Calcein-AM were added to HMrSV5 cells treated with CM from SGC-7901 cells (D) or HGC-27 cells (E) or with different *LGALS1* expression levels (Scale bars, 100 μm) (*n* = 3). (F and G) Mean IODs of SGC-7901 cells (F) and HGC-27 cells (G) adherent to HMrSV5 cells (*n* = 3). Data are represented as mean ± SD.∗∗*p* < 0.01, NS, *p* > 0.05.

Then, we utilized IF to detect the protein expression of TGF-β1 and *p*-Smad2/3 in the peritoneal tissues of 6 GC patients with peritoneal MMT and another 6 GC patients without peritoneal MMT, and quantitatively analyzed the target proteins via Image-Pro Plus software (Figures 5A and 5B). The relative fluorescence of TGF-β1 in the group with MMT was 1.375 ± 0.121, which was significantly higher than that in the group without MMT (0.613 ± 0.057) (Student’s *t* test, all *p* < 0.01, Figure 5C). The relative fluorescence of *p*-Smad2/3 in the group with MMT (1.146 ± 0.141) was significantly higher than that in the group without MMT (0.549 ± 0.026, *p* < 0.01, Figure 5C). Our findings suggest that the activation of the TGF-β/Smad signaling pathway in the peritoneal tissue of GC patients may be closely associated with the peritoneal MMT.

### Galectin-1 enhances GC cell adhesion to HPMCs through the TGF-β/Smad signaling pathway

We employed adhesion experiments to simulate GCPM *in vitro*, so as to elucidate the promoting effect of galectin-1 on GCPM and its mechanism (Figures 5D and 5E). Fluorescence examination showed that the integral optical density (IOD) of SGC-7901 cells adhering to HPMCs cells treated with SGC-7901 CM-OE-*LGALS1* was 2742.451 ± 186.253, which was significantly higher than that of the CM-SGC-7901 group (1080.749 ± 134.998) and CM-OE-NC group (981.871 ± 11.992) (one-way ANOVA, *p* < 0.01, Figure 5F). Additionally, the IOD of SGC-7901 cells adhering to CM-OE*-LGALS1*-treated HMrSV5 cells was restored by ITD1 (Student’s *t* test, Figure 5F). CM from HGC-27 was used to treat HMrSV5 cells to repeat the adhesion experiment, the results of the replication experiment were consistent with those with CM from SGC-7901 cells (Figure 5G). Collectively, galectin-1 promotes the adhesion of GC cells to HPMCs, and this promoting effect can be reversed by blocking the TGF-β/Smad signaling pathway, suggesting a TGF-β/Smad-dependent mechanism.

### Galectin-1 promotes GCPM through the TGF-β/Smad signaling pathway

**Figure 6:**
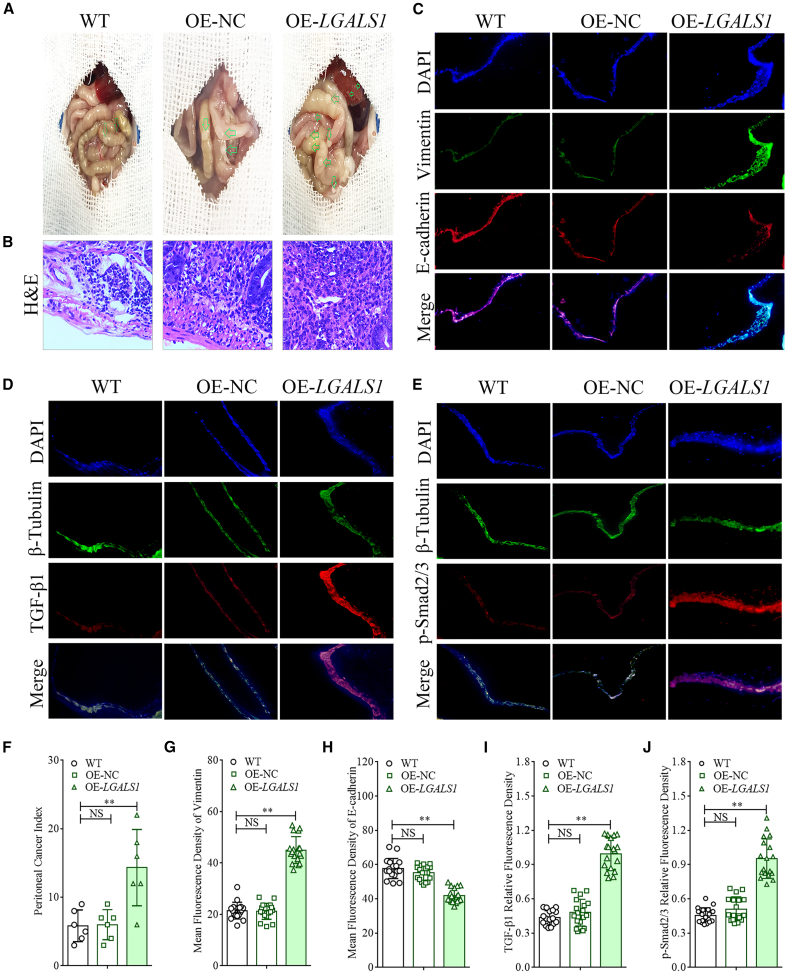
Galectin-1 promotes GCPM through the TGF-β/Smad signaling pathway (A) Representative images of the GCPM animal model established in this study. (B) H&E staining confirmed that the peritoneal nodules were metastatic carcinomas (×400 magnification). (C–E) Representative immunofluorescence images of E-cadherin and vimentin (C), TGF-β1 (D) and *p*-Smad2/3 (E) in the peritoneum of model animals (×400 magnification). (F) The PCI of mice in different groups (*n* = 6). (G and H) The mean fluorescence density of vimentin and E-cadherin (*n* = 6). (I and J) The relative fluorescence density of TGF-β1 and *p*-Smad2/3 (*n* = 6). Data are represented as mean ± SD. ∗∗*p* < 0.01, NS, *p* > 0.05.

BALB/c athymic nude mice were utilized to establish a carcinomatosis model to elucidate the promoting effect of galectin-1 on GCPM *in vivo* and explore its molecular mechanism. The results revealed that the peritoneal carcinomatosis index (PCI) in the OE-*LGALS1* group (14.333 ± 5.088) was significantly higher than that in the wild-type (WT) group (5.833 ± 2.115) and OE-NC group (6.000 ± 2.000) (one-way ANOVA, *p* < 0.01, Figures 6A and 6F). H&E staining confirmed that the peritoneal nodules were metastatic carcinomas (Figure 6B).

To determine whether galectin-1 promotes peritoneal MMT through the TGF-β/Smad signaling pathway *in vivo*, immunofluorescence was used to detect the expression of E-cadherin and vimentin in the peritoneum of model animals, and the mean fluorescence density of the target proteins in each group was compared. The statistical results revealed that the mean fluorescence density of vimentin in the OE-*LGALS1* group was 45.063 ± 5.055, which was significantly higher than that in the WT group (21.468 ± 3.117) and OE-NC group (21.212 ± 12.635) (one-way ANOVA, *p* < 0.01, Figures 6C and 6G).The mean fluorescence density of E-cadherin in the OE-*LGALS1* group (41.919 ± 10.864) was significantly lower than that in the WT group (57.734 ± 5.178) and OE-NC group (55.139 ± 7.814) (one-way ANOVA, *p* < 0.01, Figures 6C and 6H).

We utilized immunofluorescence to detect the expression of the TGF-β1 and *p*-Smad2/3 in the peritoneal tissues of different groups of model mice, with β-Tubulin used as an internal reference to analyze the relative fluorescence density of the target proteins. The relative fluorescence of TGF-β1 in the OE-*LGALS1* group (0.995 ± 0.132) was significantly greater than that in the WT group (0.435 ± 0.059) and OE-NC group (0.480 ± 0.114) (one-way ANOVA, *p* < 0.01, Figures 6D and 6I). The relative fluorescence of *p*-Smad2/3 in the OE-*LGALS1* group (0.952 ± 0.168) was significantly greater than that in the WT group (0.457 ± 0.063) and OE-NC group (0.512 ± 0.102) (one-way ANOVA, *p* < 0.01, Figures 6E and 6J).

**Figure 7:**
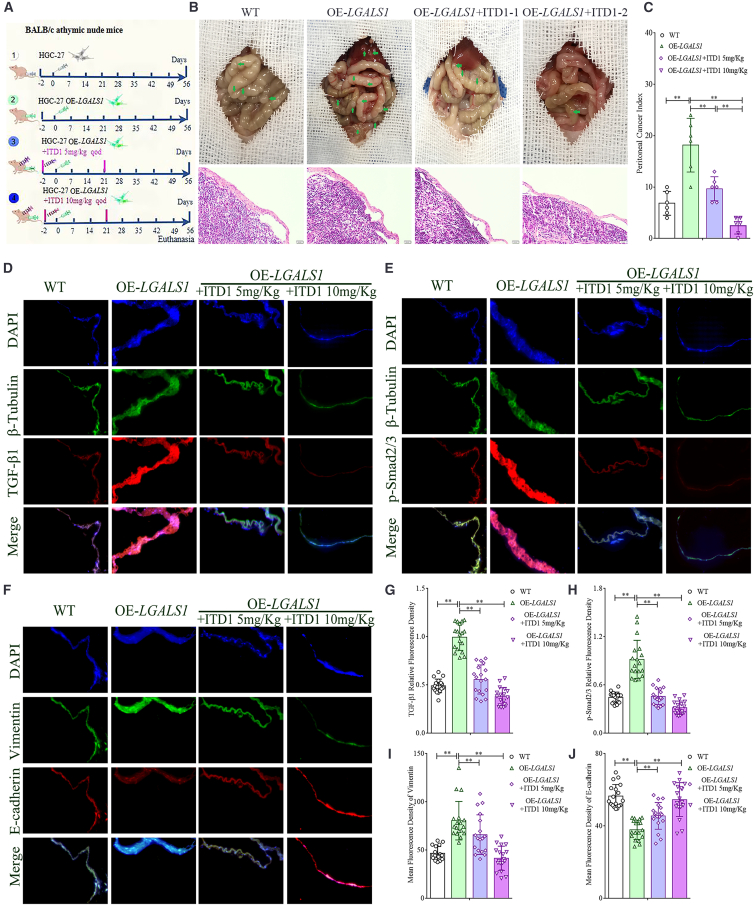
Inhibition of TGF-β1 attenuates peritoneal MMT and suppresses GCPM (A) Schematic representation of the carcinomatosis models. (B) Representative gross images of peritoneal carcinomatosis and corresponding H&E staining (×400 magnification). (C) PCI across experimental groups (*n* = 6). (D–F) Representative immunofluorescence images showing expression of TGF-β1, *p*-Smad2/3, and E-cadherin/vimentin in peritoneal tissues from model mice (×400 magnification). (G and H) The relative fluorescence density of TGF-β1 and *p*-Smad2/3 (*n* = 6). (I and J) Mean fluorescence density of vimentin and E-cadherin (*n* = 6). Data are represented as mean ± SD. ∗∗*p* < 0.01.

To further confirm the mechanistic role of galectin-1 in promoting GCPM, we administered the TGF-β1 inhibitor ITD1 in the carcinomatosis model, and the experimental timeline and treatment regimen are summarized in Figure 7A. Consistent with our hypothesis, ITD1 treatment significantly reduced the PCI in a dose-dependent manner (Student’s *t* test, all *p* < 0.01, Figures 7B and 7C). Immunofluorescence analysis further demonstrated that ITD1 effectively suppressed the expression of TGF-β1 (Student’s *t* test, all *p* < 0.01, Figures 7D and 7G) and *p*-Smad2/3 in peritoneal tissues of OE-*LGALS1* group (Student’s *t* test, all *p* < 0.01, Figures 7E and 7H). Moreover, ITD1 reversed the *LGALS1*-induced downregulation of E-cadherin and upregulation of vimentin in peritoneal tissues (Student’s *t* test, all *p* < 0.01, Figures 7F, 7I and 7J), thereby attenuating the pathological process of MMT. Our results confirm that galectin-1 promotes the formation of GCPM in animal models by promoting peritoneal MMT through activating the TGF-β/Smad signaling pathway.

## Discussion

This study investigated the regulatory role of galectin-1 in the GCPM. To the best of our knowledge, the present study is the first to reveal that galectin-1 derived from GC tissue facilitates PM by inducing MMT in PMCs. Our data indicated that galectin-1 expression is closely associated with the GCPM. Galectin-1 promotes the PM of cancers, which has been previously reported in ovarian cancer.^19^^,^^20^ In our previous study, we reported that galectin-1 promotes GCPM by enhancing peritoneal collagen deposition.^21^ Accumulating evidence has demonstrated that MMT in PMCs represents a critical prerequisite for PM.^22^^,^^23^ Through MMT, PMCs attain a stronger capacity for invasion and adhesion to cancer cells and acquire the ability to synthesize inflammatory and angiogenic factors, such as fibroblasts and vascular endothelial growth factors, which exert growth-promoting effects on cancer cells.^23^^,^^24^^,^^25^

MMT is the outcome of complex cellular reprogramming, and diverse stimuli can activate multiple signaling cascades. Among these regulatory networks, the TGF-β and WNT pathways are widely recognized as canonical pathways governing MMT.^26^^,^^27^^,^^28^ In the present study, ELISA was performed to examine the levels of TGF-β1, WNT5a, and WIF1 proteins in CM under different *LGALS1* expression status. Results demonstrated that the expression of TGF-β1 was significantly altered between groups, whereas no significant differences were observed in WNT pathway-related proteins. Accordingly, TGF-β1 is identified as a key mediator in galectin-1-induced MMT in PMCs.

Galectin-1 is one of the 15 members of the beta-galactose binding lectin family. Among them, galectin-1 and galectin-3 seem to be the principal contributors to cancer biology and thereby have attracted significant research attention. As a cytoplasmic protein, galectin-1 is synthesized on cytoplasmic ribosome and secreted extracellularly from the nucleus toward the medial portion of the cell membrane. Secretion occurs through nonclassical mechanisms involving direct translocation across the plasma membrane. Previous studies have predominantly investigated galectin-1 expression by CAFs within the tumor microenvironment and its subsequent modulation of GC cell behavior.^17^^,^^18^ In contrast, the present study focuses on galectin-1 entry into target cells and examines how galectin-1-expressing GC cells influence PMCs—particularly through the induction of MMT. To this end, GC cell lines were selected as the primary experimental models. Galectin-1 can activate the TGF-β/Smad signaling pathway to regulate the progression of benign and malignant disorders; for instance, it mediates the galectin-1/TGF-β/EMT pathway to regulate retinal pigment epithelial cell fibrosis in diabetic retinopathy,^29^ and induces EMT in GC and pancreatic cancer.^14^^,^^30^ Hence, we propose and verify the scientific hypothesis that galectin-1 promotes peritoneal MMT to induce GCPM through the TGF-β/Smad signaling pathway. Notably, while our findings indicate that galectin-1 upregulates TGF-β1 in GC cells and thereby promotes peritoneal MMT, whether this effect is mediated through malignant ascites or systemic circulation remains to be elucidated. Future investigations will address this gap by quantifying TGF-β1 levels in intraoperative peritoneal lavage fluid and matched peripheral blood samples.

In our study, 18 out of 42 patients with GC presented with peritoneal MMT. Galectin-1 levels in primary GC tissues were significantly higher in the peritoneal MMT group than in the group without peritoneal MMT, indicating that galectin-1 expression in GC tissues is associated with peritoneal MMT.*In vitro* cell experiments showed that CM derived from *LGALS1*-overexpressing GC cells significantly increased the expression of *VIM* mRNA and vimentin protein in HPMCs, while decreasing the expression of *CDH1* mRNA and E-cadherin protein. These results suggest that galectin-1 induces MMT in HPMCs, a finding that has not been previously reported.

Next, we investigated the molecular mechanism by which galectin-1 promotes MMT in HPMCs. ELISA results showed that TGF-β1 was highly expressed in the CM of *LGALS1*-overexpressing GC cells. Western blot analysis confirmed that TGF-β1 and *p*-Smad2/3 proteins were significantly upregulated in HPMCs treated with CM from *LGALS1*-overexpressing GC cells. IF staining of clinical specimens further verified that the expression levels of TGF-β1 and *p*-Smad2/3 were significantly higher in peritoneal tissues exhibiting MMT than in those without MMT.

In addition to TGF-β isoforms, the TGF-β superfamily comprises activins, inhibins, Müllerian-inhibiting substance (MIS) and bone morphogenetic proteins (BMPs).^31^ In mammals, three TGF-β isoforms have been identified: TGF-β1, TGF-β2, and TGF-β3. TGF-β is widely expressed in diverse normal tissues and cells, including osteoblasts, kidney, bone marrow, and hematopoietic cells. Among these isoforms, TGF-β1 exhibits the highest expression level in human platelets and mammalian bone tissue. Genes associated with the TGF-β signaling pathway are frequently upregulated in tumor tissues, and TGF-β mRNA is detectable in nearly all tumor cell types.^32^^,^^33^ TGF-β1 initiates intracellular signaling by binding to specific cell surface receptors, in which Smad family proteins function as central intracellular mediators. The Smad family is classified into three groups: inhibitory Smads (I-Smads: Smad6 and Smad7), the common-mediator Smad (Co-Smad: Smad4), and receptor-regulated Smads (R-Smads: Smad1, 2, 3, 5, and 9). In the canonical TGF-β signaling cascade, TGF-β exerts its biological effects through transmembrane type I and type II serine/threonine kinase receptors. TGF-β first binds to the type II receptor, which then recruits and phosphorylates the type I receptor.^31^ The activated type I receptor subsequently phosphorylates R-Smads. Phosphorylated R-Smads form a heteromeric complex with Smad4, which translocates into the nucleus to regulate the transcription of target genes involved in tumor promotion or suppression.^31^^,^^34^

Previous studies have reported similar effects of *LGALS1* on the regulation of TGF-β.^14^^,^^30^^,^^35^ and it is not uncommon for TGF-β/Smad to regulate the peritoneal MMT.^24^^,^^36^ We used the TGF-β1-specific blocking agent ITD1 to perform rescue experiments and found that the upregulated protein expression of TGF-β1 and *p*-Smad2/3 in HPMCs treated with CM from *LGALS1*-highly expressing GC cells could be blocked by ITD1. *In vitro* adhesion experiments simulating PM showed that treatment of PMCs with CM from *LGALS1*-overexpressing GC cells significantly enhanced the adhesion capacity of HPMCs to GC cells, and this effect was abrogated by ITD1. Thus, *LGALS1* enhances the adhesion ability of PMCs to GC cells through the TGF-β/Smad signaling pathway. MMT is an important mechanism that enhances the adhesion of PMCs, which has been widely reported in ovarian cancer and GCPM.^22^^,^^37^^,^^38^^,^^39^ *In vivo* studies further confirmed that ITD1 effectively reversed *LGALS1*-overexpression-driven peritoneal MMT and GCPM. These findings collectively confirm that galectin-1 promotes MMT in PMCs via the TGF-β/Smad pathway.

In animal model experiments, we employed an intraperitoneal injection model to directly inoculate tumor cells into the abdominal cavity. This approach bypasses early metastatic steps—such as primary tumor invasion and intravasation—thus allowing us to specifically focus on the peritoneal dissemination phase. Although it does not fully replicate the entire metastatic cascade, the model reliably recapitulates key pathological features of diffuse PM, including ascites and peritoneal nodule formation. We confirmed that *LGALS1* significantly promoted PCI in the mouse model, and ITD1 effectively reversed *LGALS1*-driven peritoneal MMT and GCPM. *In vivo*, upregulation of intracellular TGF-β1 can induce the deposition of collagen and fibronectin via the Smad signaling pathway, leading to peritoneal fibrosis.^40^ In this study, we did not perform Masson staining to analyze collagen deposition in the peritoneum of model mice, but immunofluorescence staining revealed that the peritoneum of mice in the *LGALS1*-overexpressing group was significantly thickened at the pathological level. The present investigation focused on changes in the metastatic “soil” rather than *LGALS1*-driven regulation of the tumor “seeds.” Subsequent investigations should therefore evaluate the influence of *LGALS1* on GC cell proliferation and its specific role in GCPM, for instance, through Ki-67 staining and other analyses of metastatic nodules in model animals. Our research further shows that blockade of the TGF-β/Smad signaling pathway can reverse peritoneal MMT and inhibit metastatic progression. An important unanswered question is whether this therapeutic effect translates into a survival advantage for the host. Addressing this limitation by assessing survival outcomes in model animals will be a crucial next step. Notably, this study was initiated based on the clinical association between elevated galectin-1 expression and increased GCPM. To model this clinical phenomenon, we generated *LGALS1*-overexpressing GC cell lines. A key limitation is that we did not investigate the reversibility of the *LGALS1*-overexpressing phenotype. Specifically, it remains to be tested whether *LGALS1* knockdown can suppress MMT in PMCs and inhibit GCPM, which will be a priority for our future mechanistic validation.

In summary, our study demonstrates that tumor-derived galectin-1 in the GC microenvironment promotes peritoneal MMT via the TGF-β/Smad signaling pathway, thereby accelerating the progression of GCPM. These findings provide mechanistic insights into the pathogenesis of GCPM, identify galectin-1 as a potential therapeutic target, and have important implications for the clinical prevention and treatment of GCPM. We further propose that TGF-β1 may trigger MMT in PMCs through ascitic fluid or systemic circulation, and that galectin-1 could disseminate hematogenously to the peritoneum, directly upregulating TGF-β1 expression in the peritoneal microenvironment and thereby facilitating MMT. Collectively, our results highlight galectin-1 as a promising therapeutic target and suggest that targeting its downstream TGF-β/Smad signaling pathway represents a viable strategy for the prevention and treatment of GCPM.

### Limitations of the study

Despite the promising findings, several limitations inherent to the present study should be acknowledged. First, the sample size of clinical GC tissues used for mechanistic analyses remains relatively small, which may restrict the statistical power and generalizability of our conclusions. Second, the expression levels of TGF-β in paired ascitic fluid and peripheral blood were not determined in this study; thus, its potential systemic or peritoneal microenvironmental contributions could not be further evaluated. Most importantly, the precise molecular cascade through which galectin-1 promotes TGF-β upregulation in primary GC tissues, as well as the long-distance paracrine or humoral mechanisms by which these signals modulate the metastatic preconditioning of the peritoneum, have not been fully delineated in the current study.

## Resource availability

### Lead contact

Further information and requests for resources should be directed to and will be fulfilled by the lead contact, Xiaolan You (006586@yzu.edu.cn).

### Materials availability

This study did not generate new unique reagents.

### Data and code availability

The original figures of microscopy images and Uncropped immunoblotting data are uploaded to Mendeley Data (https://data.mendeley.com/datasets/rzkx72y56f/1). This article does not report original code. Any additional information required to reanalyze the data reported in this article is available from the lead contact upon request.

## Acknowledgments

This work was supported by the Medical Research Project of Jiangsu Health Commission, China (M2024015), Science and Technology Support Program (Social Development) Project of Taizhou City, China (TS202416), the Taizhou sixth phase 311 talent training project (RCPY202421), Nanjing Medical University Taizhou Clinical Medical College Research Project (TZKY20250208). The authors would like to thank Dou Rongrong, Liu Fuxing, and Zhu Xiaowei, Department of Pathology, Taizhou People’s Hospital Affiliated with Nanjing Medical University, for HE and IHC evaluation, and thanks for Figdraw (https://www.figdr.aw.com) for drawing Graphical abstract.

## Author contributions

X.Y., C.H., and X.S. conceived and designed this study. Z.J., C.H., Q.D., X.S. and H.W. performed experiments, analyzed data, and prepared the figures. X.Y., C.H., Z.J., X.S., Z.C., and G.L. analyzed data. X.Y. and Z.J. wrote the first version of the manuscript. X.Y., X.S., Q.D., and X.Z., revised the manuscript. X.Y. funded this research. All authors have read and approved the final manuscript.

## Declaration of interests

The authors declare no competing interests.

## STAR★Methods

### Key resources table

**Table:** 

REAGENT or RESOURCE	SOURCE	IDENTIFIER
**Antibodies**		

Mouse monoclonal antibody to beta-Tubulin	Affinity	Cat#T0023; RRID: AB_2813772
Fluor-488-conjugated goat polyclonal anti-mouse IgG (H + L)	Affinity	Cat#S0017; RRID: AB_2846212
Rabbit polyclonal antibody to E-cadherin	Affinity	Cat#AF0131; RRID: AB_2833315
Mouse monoclonal antibody to Vimentin	Affinity	Cat#BF8006; RRID: AB_2847777
Fluor-594-conjugated goat polyclonal anti-rabbit IgG (H + L)	Affinity	Cat#S0006; RRID: AB_2843436
Rabbit monoclonal antibody to TGF-β1	Abcam	Cat#ab170847
Rabbit polyclonal antibody to GAPDH	Abcam	Cat#ab9485; RRID: AB_307275
Rabbit monoclonal antibody to Galectin-1	Abcam	Cat#ab138513; RRID: AB_2894851
Rabbit polyclonal antibody to TGF-β1	Bioss	Cat#bs-0086R; RRID: AB_10856457
Rabbit polyclonal antibody to *p*-Smad2/3	Bioss	Cat#bs-8853R
Rabbit polyclonal antibody to Vimentin	Bioss	Cat#bs-0756R; RRID: AB_10855343
Rabbit polyclonal antibody to E-cadherin	Bioss	Cat#bs-10009R; RRID: AB_2924843
HRP-conjugated rabbit polyclonal goat anti-rabbit IgG	Bioss	Cat#bs-0295G-HRP; RRID: AB_10923693

**Bacterial and virus strains**		

Lentiviral vector expressing *LGALS1* (GV358; Ubi-MCS-3FLAG-SV40-EGFP-IRES-puromycin)	GeneChem Co., Ltd.	N/A
Lentiviral negative control vector (GV358; Ubi-MCS-3FLAG-SV40-EGFP-IRES-puromycin)	GeneChem Co., Ltd.	N/A

**Biological samples**		

Human GC tissue samples	The Affiliated Taizhou People’s Hospital of Nanjing Medical University	Ethics approval number: KY 2024-095-01
Human peritoneal tissue samples from GC patients	The Affiliated Taizhou People’s Hospital of Nanjing Medical University	Ethics approval number: KY 2024-095-01
Mouse peritoneal metastatic carcinoma tissues	This study	Ethics approval number: YZU-EC-202406030
Mouse peritoneal tissues from GCPM animal model	This study	Ethics approval number: YZU-EC-202406030

**Chemicals, peptides, and recombinant proteins**		

ITD1	Beyotime	Cat#SF7899
DAPI	Beyotime	Cat#C1006
Calcein AM	Beyotime	Cat#C2012
RPMI 1640 medium	Gibco	Cat#22400089
DMEM medium	Gibco	Cat#11965092
PBS	Gibco	Cat#10010023
FBS	Gibco	Cat#10099141

**Critical commercial assays**		

Human TGF-β1 ELISA	Solarbio	Cat#SEKH-0316
Human Galectin-1 ELISA	Solarbio	Cat#SEKH-0526
Human WNT5 ELISA	Chundu	Cat#CD13059
Human WIF1 ELISA	Chundu	Cat#CD15539
Reverse transcription kit	Solarbio	Cat#RP1105
qPCR kit	Solarbio	Cat#RP1100

**Experimental models: Cell lines**		

Human gastric cancer cell line SGC-7901	Beyotime Biotech, Inc.	Cat#C6795
SGC-7901 cells stably overexpressing *LGALS1*	This study	N/AN
SGC-7901 negative control stable cell line	This study	N/A
Human gastric cancer cell line HGC-27	Beyotime Biotech, Inc.	Cat#C6365
HGC-27 cells stably overexpressing *LGALS1*	This study	N/A
HGC-27 negative control stable cell line	This study	N/A
Human peritoneal mesothelial cell line MrSV5	BeNa Culture Collection	Cat#BNCC358140

**Experimental models: Organisms/strains**		

BALB/c nude mice	The Comparative Medicine Center of Yangzhou University	SCXK(Su)2022-0009
Mouse peritoneal metastasis model	This study	N/A

**Oligonucleotides**		

qPCR primers for target genes	Sangon	N/A

**Recombinant DNA**		

Lentiviral vector expressing *LGALS1*	This study	N/A

**Software and algorithms**		

GraphPad Prism	GraphPad Software	RRID: SCR_002798
IBM SPSS Statistics software	IBM Corp.	RRID: SCR_002865
ImageJ	NIH	RRID: SCR_003070
Image-Pro Plus software	Media Cybernetics	RRID: SCR_007369

### Experimental model and study participant details

#### Human participants

Human GC tissues and corresponding peritoneal tissues were collected from 42 GC patients admitted to the Department of Gastrointestinal Surgery, The Affiliated Taizhou People’s Hospital of Nanjing Medical University. The study was conducted according to the Declaration of Helsinki guidelines and was approved by the Clinical Research Ethics Committee of Taizhou People’s Hospital (KY 2024-095-01 Jiangsu, China). The detailed assessment procedures were explained to the patients and written informed consent was obtained from all patients.

#### Mice and ethics statement

Male BALB/c athymic nude mice (5 weeks old, 17–20 g) were purchased from the Comparative Medicine Center of Yangzhou University (Jiangsu, China). All animal experiments were approved by the Ethics Committee of Yang Zhou University (YZU-EC-202406030, Jiangsu, China). and performed in accordance with the ARRIVE (Animal Research: Reporting of *In Vivo* Experiments) guidelines as well as relevant institutional guidelines and regulations.

#### Cell culture

Two GC cell lines, SGC-7901 (C6795) and HGC-27 (C6365), were obtained from Beyotime Biotech, Inc., and cultured in RPMI 1640 medium supplemented with 10% (v/v) fetal bovine serum (FBS). The HPMC line HMrSV5 was acquired from BeNa Culture Collection (Beijing, China) and maintained in Dulbecco’s modified Eagle’s medium (DMEM) containing 10% (v/v) FBS. All cells were cultured at 37 °C in a humidified atmosphere with 5% CO_2_. Cells were digested with 0.25% trypsin when they reached 80% confluence.

### Method details

#### Immunohistochemistry (IHC) and IHC evaluation

Tissues used for IHC analysis were cut into 3-μm sections. All sections were dewaxed in xylene and rehydrated through a graded ethanol series, followed by antigen retrieval via boiling in 0.01 M citrate buffer (pH 6.0) for 15 min using a microwave oven. Primary antibodies recognizing galectin-1 (dilution, 1:500), E-cadherin (dilution, 1:200), and vimentin (dilution, 1:200) were used and incubated with the tissue slides overnight at 4 °C. The samples were subsequently processed in accordance with standard procedures, and two pathologists blinded to the clinical status of the patients independently evaluated the staining results. Ten fields were randomly selected from each section under a microscope at 400× magnification. The percentage of positively stained cells was scored as follows: 1 (0–5%), 2 (6–25%), 3 (26–50%), or 4 (>50%). The staining intensity was scored as 0 (negative), 1 (weak), 2 (moderate), or 3 (strong). Any discrepancies between the two pathologists were resolved by consensus discussion. The immunoreactivity score (IRS) was calculated as the product of the percentage score and the intensity score.

#### Immunofluorescence (IF)

GC cells were cultured in 24-well plates with glass coverslips and treated with conditioned medium (CM) for 72 h or with ITD1 (10 μM), a specific antagonist of the TGF-β/Smad signaling pathway, for 24 h. Cells were then fixed with 4% formaldehyde. For tissue samples, paraffin embedding was performed, and 3-μm sections were prepared, followed by antigen retrieval via pressure cooking. Cells or tissue sections were permeabilized with 1% Triton X-100 and blocked with 5% bovine serum albumin for 30 min. Primary antibodies (rabbit anti-E-cadherin, mouse anti-vimentin, rabbit anti-TGF-β1, rabbit anti-*p*-Smad2/3, and mouse anti-β-tubulin) were incubated overnight at 4 °C. Goat anti-mouse IgG Alexa Fluor 488-conjugated (Affinity, S0017) and goat anti-rabbit IgG Alexa Fluor 594-conjugated (Affinity, S0006) secondary antibodies were then applied for 1 h at room temperature. Nuclei were stained with DAPI for 10 min. Fluorescence images of cell monolayers were captured under a fluorescence microscope, and the mean fluorescence intensity of target proteins was quantified using Image-Pro Plus software.

#### Lentiviral transfection

Lentiviral vectors for *LGALS1* overexpression (OE-*LGALS1*) and negative control (OE-NC), both expressing green fluorescent protein (GFP) and the puromycin resistance gene, were purchased from Genechem Co., Ltd. (Shanghai, China). SGC-7901 and HGC-27 cells were seeded into 6-well plates at a density of 2 × 10^4^ cells/mL (2 mL per well). Lentiviral particles were added with 10 μg/mL polybrene (Sigma-Aldrich) overnight according to the optimal multiplicity of infection (MOI). After incubation at 37 °C in a 5% CO_2_ atmosphere for 12 h, the medium was replaced with fresh complete medium. Stably transduced cell lines were selected using 2 μg/mL puromycin (Sigma-Aldrich) for 48 h, and the stable transfectants were subsequently maintained in medium containing 0.5 μg/mL puromycin. The transduction efficiency was verified by qRT-PCR and western blotting at 72 h post-transduction.

#### Enzyme-linked immunosorbent assay (ELISA)

The levels of Galectin-1, TGF-β1, WNT5A, and WIF-1 in the CM of GC cells with different *LGALS1* expression levels were detected using commercially available ELISA kits. All experiments were performed strictly following the manufacturer’s instructions. For each group of GC cells (with different *LGALS1* expression levels), three independent CM samples were prepared, and each sample was assayed in triplicate.

#### qRT–PCR

Total cellular RNA was extracted from HMrSV5 cells qRT-PCR. First-strand complementary DNA (cDNA) was synthesized using an RT kit. Subsequently, quantitative real-time PCR (qRT-PCR) was performed using the iQ5 Multicolor Real-Time PCR Detection System and SYBR Green. Glyceraldehyde-3-phosphate dehydrogenase (GAPDH) was used as an internal control to calculate the relative expression levels, and the comparative threshold cycle(2^−ΔΔCt^) method was employed for quantification. All experiments were performed in independent triplicate. The primers for *CDH1*, *VIM*, and *GAPDH* are listed in Table 1.

**Table 1:** Primers used for qRT-PCR in the present study

Gene	Gene Primer sequence (5′–3′)
*CDH1*	F: CTGCTGCTCTTGCTGTTTCTTCG
	R: CTCTTCTCCGCCTCCTTCTTCATC
*VIM*	F: CCTTCGTGAATACCAAGACCTGCTC
	R: AATCCTGCTCTCCTCGCCTTCC
*GAPDH*	F: CCAGCAAGAGCACAAGAGGAAGAG
	R: GGTCTACATGGCAACTGTGAGGAG

#### Protein extraction and western blotting

Sodium dodecyl sulfate–polyacrylamide gel electrophoresis (SDS-PAGE) was used to separate proteins extracted from HMrSV5 cells. The gel plates were then cut based on the molecular weight of the target protein and transferred onto nitrocellulose membranes (GE Healthcare Life Sciences, Pittsburgh, PA, USA) for antibody hybridization. Primary antibodies against E-cadherin, vimentin, TGF-β1, *p*-Smad2/3 and GAPDH (1:2000 dilution) were utilized to probe the blots. The secondary antibody was also used at a 1:2000 dilution. West Pico Chemiluminescent Substrate (Pierce, Carlsbad, CA, USA) was used to visualize the protein bands, which were quantified via ImageJ software. All the assays were performed in triplicate.

#### Tumor cell adhesion assay

HMrSV5 cells were seeded at a density of 3×10^5^ cells per well in a 24-well plate and cultured in complete medium for 24 h. Subsequently, the medium was replaced with one containing 10% CM for another 72 h or with ITD1 (10 μM) for 24 h. After pre-treatment was completed, all culture medium was removed and cells were gently washed three times with PBS. GC cells received the same pre-treatment and were then incubated with 15 μM calcein-AM for 30 min. The GC cells were then added to the HMrSV5 cells at 1×10^5^ cells per well and incubated for 3 h. Non-adherent tumor cells were removed by washing three times with culture medium. Adherent GC cells were observed under a fluorescence microscope, and fluorescence was measured at 419 nm excitation and 514 nm emission using a spectrofluorometer. The average optical density of GC cells adhering to HMrSV5 cells was determined using Image-Pro Plus software.

#### Animal assays

Mice were housed in a specific pathogen–free (SPF) animal facility at 24 °C with 40–60% relative humidity. One week before the experiment, all mice were allowed free access to standard food and purified water for environmental adaptation. For model establishment, all animals received an intraperitoneal injection of cell suspension (1.2 × 10^7^ cells/mL, 200 μL per mouse). Initially, 18 mice were randomly divided into three groups (*n* = 6 per group): the SGC-7901 wild-type (WT) group, the SGC-7901 lentiviral negative control (OE-NC) group, and the SGC-7901 lentivirus-mediated *LGALS1* overexpression (OE-*LGALS1*) group. Another 24 mice were assigned to two groups: the HGC-27 WT group (*n* = 6) and the HGC-27 OE-*LGALS1* group (*n* = 18). Among the 18 mice in the OE-*LGALS1* group, 12 were further divided equally into two subgroups (*n* = 6 each) and received intraperitoneal injection of ITD1 at 5 mg/kg or 10 mg/kg, respectively. Administration was initiated 2 days before tumor inoculation, and injections were repeated every 3 days until 3 weeks after tumor inoculation. Eight weeks after cell inoculation, all mice were euthanized by CO_2_ inhalation and dissected. The peritoneal carcinomatosis index (PCI) was used to evaluate metastatic tumor burden. Histopathological evaluation was performed using hematoxylin and eosin (H&E) staining, and immunofluorescence analysis was also conducted.

### Quantification and statistical analysis

GraphPad Prism 6.0 was used for graph generation in this study. Statistical analyses were conducted using IBM SPSS Statistics, version 25 (IBM Corp., Armonk, NY, USA). Continuous variables with a normal distribution are expressed as the mean ± standard deviation (SD). For statistical comparisons, Student’s *t* test was used for pairwise comparisons between two groups, one-way analysis of variance (ANOVA) followed by Dunnett’s post hoc test was used for multiple comparisons to compare each group with the control group, and the chi-square test was applied for comparisons of categorical variables. Differences were considered statistically significant at *p* < 0.05 (∗) and *p* < 0.01 (∗∗).
